# Environmental conditions but not nest composition affect reproductive success in an urban bird

**DOI:** 10.1002/ece3.7234

**Published:** 2021-03-12

**Authors:** Pablo Capilla‐Lasheras, Blanca Bondía, José I. Aguirre

**Affiliations:** ^1^ Institute of Biodiversity, Animal Health and Comparative Medicine University of Glasgow Glasgow UK; ^2^ Department of Biodiversity, Ecology and Evolution Universidad Complutense de Madrid Madrid Spain

**Keywords:** feathers, nest composition, rainfall, reproductive success, tree sparrow

## Abstract

Adjusting the composition of their nests, breeding birds can influence the environmental conditions that eggs and offspring experience. Birds often use feathers to build nests, presumably due to their insulating properties. The amount of feathers in nests is often associated with increased nestling survival and body condition. However, it is unclear whether these putative beneficial effects of adding feathers to nests are relevant in a wide range of environmental conditions. Here, we combine data on weather conditions and feathers in nests (i.e., nest composition) to investigate their relative contribution to reproductive success in the Eurasian tree sparrow (*Passer montanus*). Specifically, we investigate whether the effect of weather conditions on breeding success is modulated by the amount of feathers added to the nest. We found a strong negative effect of rainfall on the number of nestlings that successfully fledged per breeding attempt, but this negative effect was not mitigated by the amount of feathers in nests. We also found that the amount of feathers in nests varied along the breeding season, with nests containing more feathers early in the breeding season, when temperatures were lower. Despite considerable variation in nest composition, our results do not suggest an important role of feathers in nests protecting eggs or nestling tree sparrows against fluctuations in environmental conditions.

## Graphical abstract

Adjusting the composition of their nests, birds can influence the environmental conditions that eggs and offspring experience. We combined data on weather conditions and nest composition to investigate their relative contribution to reproductive success of the Eurasian tree sparrow (*Passer montanus*). Our results suggest that, despite considerable variation in nest composition, the use of feathers in nests is not particularly beneficial to mitigate negative rainfall effects on the reproductive success of tree sparrows.

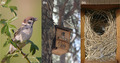

## 1 INTRODUCTION

Environmental conditions during development are critical for survival and can affect the expression of behavioral and life‐history traits (Lindström, 1999; Metcalfe & Monaghan, 2001). Changes in external conditions are particularly relevant for oviparous species (e.g., reptiles and birds). In these species, embryos develop inside an egg and are potentially exposed to environmental shifts that can involve suboptimal conditions for development (Ackerman & Lott, 2004; Ar & Sidis, 2002). In order to reduce such environmental variation, egg‐laying species, and birds in particular, have evolved a suite of incubation strategies and behavioral adaptations (Deeming, 2002a; Deeming & Ferguson, 1991).

In birds, eggs are often incubated by breeding individuals in nests (Deeming, 2002a, 2016). The incubating bird forms a functional unit with its nest, providing appropriate conditions for the development of embryos (Deeming, 2016). The functional properties of the bird‐nest unit can be shaped by the behavior of the incubating individual (Cooper & Voss, 2013; Deeming, 2002b) and also by the structure and composition of the nest (Hansell, 2000; Hilton et al., 2004). Avian nests vary in composition both among species (Collias, 1997; Hansell, 2000) and within species (e.g., due to adaptive plasticity; Deeming et al., 2012; Mainwaring & Hartley, 2008; Mainwaring et al., 2012; McGowan et al., 2004). Such variation is thought to have an adaptive role in buffering the impact of adverse environmental conditions on the development of embryos and nestlings (Deeming et al., 2012; Mainwaring & Hartley, 2008; Mainwaring et al., 2012). In general, nests built in cold conditions have better thermal insulation (Deeming et al., 2012), for example, at the beginning of the breeding season in temperate habitats (Mainwaring & Hartley, 2008), at high latitudes (Mainwaring et al., 2012, 2014), or at high altitudes (Heenan et al., 2015; but also see Schöll & Hille, 2014). There is, indeed, experimental evidence showing that birds can plastically adjust nest composition in response to shifting environmental conditions (Campbell et al., 2018), which is also supported by correlative studies (Deeming et al., 2012; McGowan et al., 2004).

Materials used by birds to build their nests differ in the thermal properties that they confer (Hilton et al., 2004). In particular, feathers provide high insulation (Hilton et al., 2004; Pinowski et al., 2006; Windsor et al., 2013), which is presumably one reason why feathers are often found at the top layer of nests (i.e., nest cup) in close contact with eggs (Hansell, 2000). Accordingly, nests of several passerine birds contain more feathers when temperatures are colder (e.g., within species, throughout their breeding season; Liljesthröm et al., 2009; Mainwaring & Hartley, 2008; Mainwaring et al., 2012; McGowan et al., 2004). For example, blue tits (*Cyanistes caeruleus*) and long‐tailed tits (*Aegithalos caudatus*) include more feathers in their nests at the beginning of the breeding season, when temperatures are low, than later in the season when temperatures are higher (Mainwaring & Hartley, 2008; McGowan et al., 2004). The benefits for offspring of adding feathers to the nest are thus predicted to be influenced by variation in environmental conditions. As such, the amount of feathers in nests may have strong positive effects on offspring survival under harsh environmental conditions (e.g., in particularly cold conditions), but weak or no effects in milder environmental conditions. Experimental studies in which the amount of feathers in nests was reduced or increased suggest that feathers have positive effects on the development and survival of nestlings (Dawson et al., 2011; Lombardo et al., 1995; Stephenson et al., 2009; Winkler, 1993). However, there is limited knowledge of the effects of nesting feathers on offspring survival under different environmental conditions (e.g., harsh and mild conditions).

**FIGURE 1:**
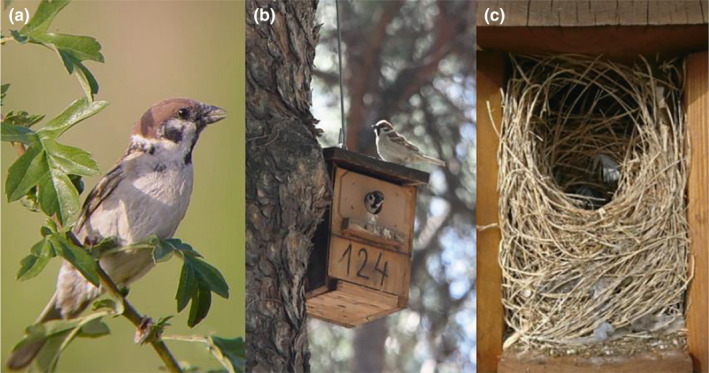
Eurasian tree sparrow and its nest in our study population. (a) Adult tree sparrow (*Passer montanus*). (b) Nest‐box type used in this study, occupied by a pair of tree sparrows. (c) Tree sparrow nest built inside a nest box. Tree sparrows use grass and tweaks to build the nest and then lined it with feathers

Here, we use detailed data on nest composition, weather conditions, and breeding success to investigate whether the amount of feathers in nests improves the reproductive success of the Eurasian tree sparrow (*Passer montanus*; Figure 1) and whether detrimental weather effects on breeding success are mitigated by amount of feathers in nests. Tree sparrows show high variation in nest composition, with nests varying in the amount of grasses, human‐made materials, and feathers that they contain (Figure 1c; Barlow et al., 2020). In our study population (in a Mediterranean area), tree sparrows have a long breeding season, from April to August. Thus, weather conditions during incubation and nestling growth markedly vary within breeding seasons (i.e., between subsequent breeding attempts of a breeding pair within the same breeding season; see Figure S1) as well as across different breeding seasons. We first evaluate whether environmental conditions have a negative effect on reproductive output in this population of tree sparrows. Then, we investigate whether variation in the amount of feathers in nests is associated with breeding success through the mitigation of negative weather effects on egg and nestling survival. Specifically, if feathers improve nest microclimate, we predict a positive effect of the amount of nesting feathers on egg and nestling survival under harsh weather conditions. We also predict a weak positive effect of feathers, or even no effect, under benign weather conditions. Finally, we investigate temporal variation in the amount of feathers in nests and whether their quantity correlates with environmental conditions. If the use of feathers in nests represents a strategy to cope with adverse environmental conditions in our study population, we predict a negative association between the amount of feathers in nests and air temperature, and a positive association between the amount of feathers in nests and rainfall during reproduction.

## 2 METHODS

### 2.1 Monitoring of breeding parameters

Fieldwork was carried out in Madrid City (Central Spain). During the breeding seasons of 2012 and 2013 (March to August), a total of 233 standardized wooden nest boxes (dimensions: 220 × 140 × 140 mm; entrance diameter: 28 mm; Figure 1b and c) were deployed and routinely monitored. Nest boxes were placed in tree branches hanging on a metal hook at a maximum height of three meters at three urban locations: the Oeste Park (40º26'03'' N, 3º43'46''W), the Alfonso XIII Botanical Garden (40º26'55''N, 3º43'43''W) and the area around the Faculty of Biology of the Complutense University (40º26'55''N, 3º43'43''W). These three study sites are located in urban matrices, in which green areas are intersected with roads and pedestrian streets (Fernández‐juricic, 2000).

We checked nest boxes weekly to detect new clutches. Once a new clutch was detected, we monitored its eggs until clutch/brood failure or fledging. For each clutch, we recorded clutch completion dates (i.e., date in which the whole clutch had been laid, assuming one egg was laid per day), egg survival to hatching and nestling survival to fledging. Nestlings were ringed after a minimum of seven days of life with uniquely identifiable bird rings (handling and ringing were carried out by PC‐L and JIA under permission of the Spanish Ringing Scheme and Madrid authorities). After fledging, nest boxes were checked for dead hatchlings, thereby enabling us to record the total number of nestlings successfully fledged (i.e., nestling survival). Tree sparrows incubate eggs for 10–15 days, and their nestlings fledge after 14–20 days of life, reaching control of their body temperature after 10 days of life (Barlow et al., 2020). In total, we monitored 159 breeding attempts (64 in 2012 and 95 in 2013), 108 first clutches and 51 s clutches in 75 different nest boxes. In our population of tree sparrows, we detected third clutches, but their frequency was low (*N* = 11 third broods across two years of study) and they experienced very low variation in environmental conditions. Therefore, we did not include them in our analyses. At the end of each breeding season (i.e., in August), nests were collected to assess nest composition. Weather data (daily minimum temperature and daily rainfall) were extracted from the European Climate Assessment & Data set (http://eca.knmi.nl/) for Retiro weather station (40º24'55''N, 3º41º03''W; approximately 4 km to the southeast of the study site).

### 2.2 Assessment of nest composition

After collection, nests were dried in a heater (MMM group, EcoCell) at 40ºC during at least 24 hr immediately before assessing their composition. The composition of all nests was assessed within three months after collection. Nest materials were manually separated into three different categories: feathers, anthropogenic materials (mainly plastic sheets), and other organic material (mainly grass and leaves). We were mainly interested in the effect of feathers on reproductive success, but we quantified full nest composition (Table S1). Feathers, and other nest materials (Table S1), were weighed to the nearest 0.01 gram using an electronic balance (A&D, GF‐200Mg). The assessment of nest composition was carried out blindly to information on reproductive success by PC‐L and BB in 2012 and 2013, respectively. Both observers followed the same protocol: Nests were thoroughly dissected until no nest materials remained uncategorized. Nest composition data were collected for a total of 66 breeding attempts randomly chosen (31 in 2012 and 35 in 2013).

### 2.3 Statistical analysis

#### 2.3.1 Environmental effects on reproductive success

We first investigated how environmental conditions affected reproductive success of urban tree sparrows. For every breeding attempt, we calculated the total amount of rainfall and the mean daily minimum temperature during incubation (up to 12 days after clutch completion day) and nestling phase (between 12 and 22 days after clutch completion day, comprising the first 10 days of a nestling's life). In our analyses, we use daily minimum temperature instead of daily mean temperature to capture the coldest end of the daily variation in temperature experienced by eggs and nestlings.

We used generalized linear mixed models (GLMM) to explain variation in different breeding parameters. Number of eggs that hatched (i.e., egg survival to hatching; *N* = 157 clutches) and number of nestling that fledged (i.e., nestling survival to fledging; *N* = 157 broods) were analyzed using two GLMMs with Poisson error structure (using a log link function). These models included site (a three‐level factor) and nest‐box identity (a factor with 75 levels) as random intercept terms. Clutch completion date (days after 1 January), clutch size, rainfall, and mean minimum temperatures during incubation (for both egg and nestling survival analyses) or during the nestling phase (for the analysis of nestling survival only) were included as fixed effects. Temperature and rainfall variables, and clutch completion date were included as linear and quadratic terms. Breeding year (two‐level factor) and brood order (two‐level factor for first or second clutches in a given nest box) were also included as fixed effects to control for additional sources of variation that might confound our results regarding weather effects on reproductive success.

#### 2.3.2 Mitigation of weather effects on reproductive success by nest materials

Using the subsample of breeding attempts with nest composition information (*N* = 66 breeding attempts—that is, last breeding attempt in a given nest box before we collected and assessed nest composition), we investigated whether nesting feathers reduced the negative effects of weather conditions on reproductive success. We analyzed variation in the number of eggs that hatched (*N* = 66 breeding attempts) and number of nestlings that fledged (*N* = 66 breeding attempts) applying those GLMMs specified above (i.e., same link function and random effect structure, and similar fixed‐effect structure), with the addition of a new set of fixed effects: weight of feather content in nests, and the interactions between the linear effect of (a) rainfall and (b) temperature, and the weight of feathers in nests. Additionally, we investigated temporal variation in the amount of feathers in nests and whether their quantity correlated with environmental conditions. To this end, we used a subset of 32 first breeding attempts (14 broods in 2012 and 18 broods in 2013). Variation in the weight of feathers in nests was explained by a linear mixed model that included site and nest‐box identity as random intercept terms. We included minimum temperatures and total rainfall during nestling rearing as explanatory variables (as environmental conditions at this reproductive stage were found to affect nestling survival—see Results). Breeding year and clutch completion date were also included in this model as fixed effects.

#### 2.3.3 General statistical procedures

The importance of every predictor explaining variation in egg survival to hatching, nestling survival to fledging, and the amount of feathers in nests was investigated using an information‐theoretic approach. For each of those three traits (i.e., dependent variables), we built a global model containing every single predictor and interaction as detailed above. Then, models with all combinations of predictors were fitted to the data and ranked based on Akaike's information criterion (AIC, Burnham, 2004). Models with a ΔAIC value lower than six were further considered (i.e., ΔAIC for the best model equals zero; for models below the top, their ΔAIC equals the difference between their own AIC and the AIC of the best model). We further reduced the top‐model set (i.e., set of models within a ΔAIC of six) by applying the nesting rule described by Richards (2008). This procedure avoids the retention of redundant combinations of fixed‐effect predictors by removing models that are more complex versions of simpler (nested) models with poorer AIC support (Arnold, 2010). When assessing the AIC value of different models, intercept‐only models were always considered. To carry out AIC comparisons, models were fitted using maximum likelihood (ML). Linear coefficients were always present in models containing quadratic coefficients. Continuous variables were standardized by mean centering and scaling by one standard deviation; therefore, model coefficients (i.e., effect sizes) are comparable across predictors. Poisson models were checked for overdispersion (by comparing their residual deviance against their residual degrees of freedom) and zero inflation; we found no indication of either. Statistical analysis was performed in R version 3.6.2. (R Core Team, 2019). We report the amount of variation explained by our models for each trait (i.e., response variable), *R* ^2^, following Nakagawa and Schielzeth (2013).

## 3 RESULTS

### 3.1 Weather effects on reproductive success

Broods experienced, on average, 19.99 mm of rainfall during the incubation period (standard deviation = 18.92; range = 0–64.3 mm) and 10.24 mm of rainfall during nestling development (*SD* = 12.51; range = 0–54.3). In these two reproductive stages, mean daily minimum temperatures were similar: 12.60°C (*SD* = 4.01°C) during incubation and 14.17°C (*SD* = 4.06°C) during nestling development (see Figure S1 for an illustration of weather conditions throughout the breeding season). First broods were laid between 15 April and 6 June (mean ± *SD* = 6 May ± 13.18 days) in 2012 and between 10 April and 10 July (mean ± *SD* = 7 May ± 18.37 days) in 2013. Second broods were laid, on average, 30 days later than first broods in 2012 (mean ± *SD* = 6 June ± 12.96 days) and 40 days later than first broods in 2013 (mean ± *SD* = 17 June ± 17.18 days).

**FIGURE 2:**
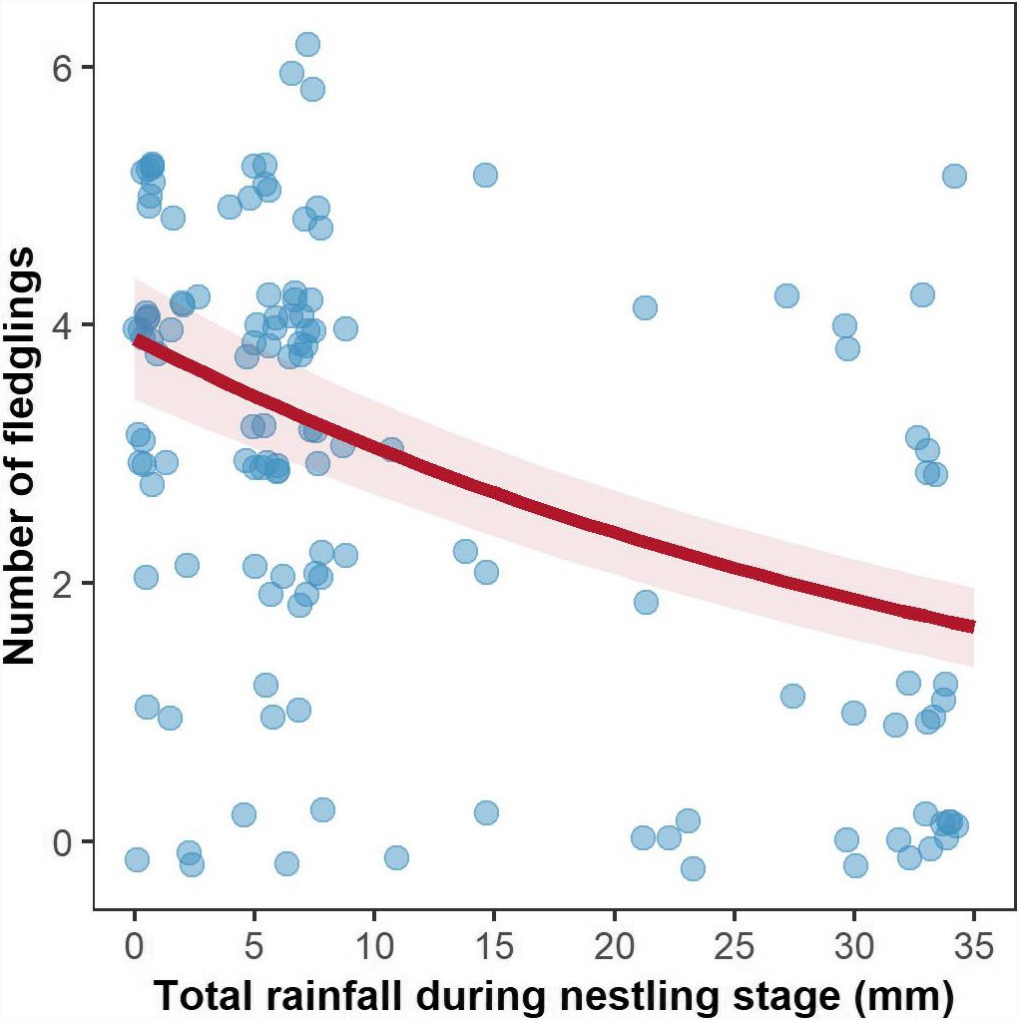
Rainfall effects on the number of nestlings that survived to fledging. The number of nestlings that survived to fledging per brood was negatively associated by total rainfall in the first ten days of life of tree sparrow nestlings. Number of nestlings that fledged (i.e., *y*‐axis) and total rainfall during the nestling stage (i.e., *x*‐axis) for 157 broods (see Table 1a). Mean ± *SE* (standard error) model predictions are illustrated in red (based on top model in Table 1a), with raw data point represented by transparent blue dots. Model predictions are averaged over the observed range of clutch sizes (2–8 eggs)

The number of nestlings that survived to fledging was strongly and negatively affected by the amount of rainfall fallen during nestling development (i.e., first ten days after hatching; Table 1a; Figure 2). Our analyses indicated a 24% reduction in the number of nestlings that survived to fledgling per 10 mm of rainfall fallen during nestling development. As expected, clutch size had a positive effect on the number of fledged nestlings (ΔAIC = 7.8; Table 1a). The top‐model set explaining variation in the number of nestlings that fledged also included effects of clutch completion date, temperature, rainfall during incubation, and breeding year, but models containing these predictors received weak statistical support (Table 1a). Environmental conditions during incubation did not predict the number of nestlings that fledged (Table 1a). We did not find important environmental effects on the number of eggs that hatched (Table 1b). Minimum temperatures and total rainfall during incubation, and clutch completion date, appeared in the top‐model set but models without these predictors received very similar statistical support (Table 1b). The number of eggs that hatched was only positively predicted by clutch size (ΔAIC = 16.4; Table 1b). Top models explaining variation in the number of nestlings that fledged and the number of eggs that hatched explained 24.57% and 15.08% of the total variation in each trait (i.e., *R* ^2^ – Nakagawa & Schielzeth, 2013).

**TABLE 1:** Environmental effects on (a) number of tree sparrow nestlings that successfully fledged and (b) on number of hatched eggs per brood

a) Number of nestlings that fledged (*N* = 157 broods)										
Intercept	Rainfall (incubation)	Rainfall (nestling phase)	Minimum temperature (nestling phase)	Minimum temperature (nestling phase)^2^	Clutch size	Clutch completion date	Breeding year^a^	*k*	AIC	ΔAIC
0.982		−0.366			0.162	−0.102		6	591.7	0.0
0.980		−0.206	0.067	−0.139	0.149			7	592.1	0.4
0.995	0.071	−0.302			0.158		0.081	7	592.3	0.6
0.997		−0.277			0.148		0.080	6	592.3	0.6
0.982	0.071	−0.321			0.170			6	592.8	1.1
0.984		−0.303			0.161			5	592.8	1.1
0.985			0.217	−0.220	0.146			6	594.2	2.5

b) Number of eggs that hatched (*N* = 157 clutches)									
Intercept	Rainfall (incubation)	Minimum temperature (incubation)	Clutch size	Brood order^b^	Clutch completion date	Clutch completion date^2^	*K*	AIC	ΔAIC
1.243	0.270	0.230	0.190		0.016	−0.111	8	590.6	0.0
1.248	0.165	0.163	0.199				6	590.9	0.3
1.290	0.091		0.200	−0.112			6	591.0	0.4
1.249			0.190				4	591.3	0.7

Note

Models within a ΔAIC value of six retained after applying the nesting rule (Richards, 2008) are shown. Model coefficients are mean‐centered and scaled by one standard deviation. *N* = sample size; *k* = number of model parameters. “Site” and “nest‐box ID” were included as random intercepts. Predictors that did not appear in any model within a ΔAIC value of six after applying the nesting rule are not presented in this table (see methods for a full list of predictors included in these models). Top models in a and b explained 24.57% and 15.08% of the total variation in the number of nestlings that fledged and the number of eggs that hatched.

^a^ Estimate for the 2012 breeding year

^b^ Estimate for 1st clutches

### 3.2 Do feathers in nests mitigate the negative effects of weather conditions on reproductive success?

**FIGURE 3:**
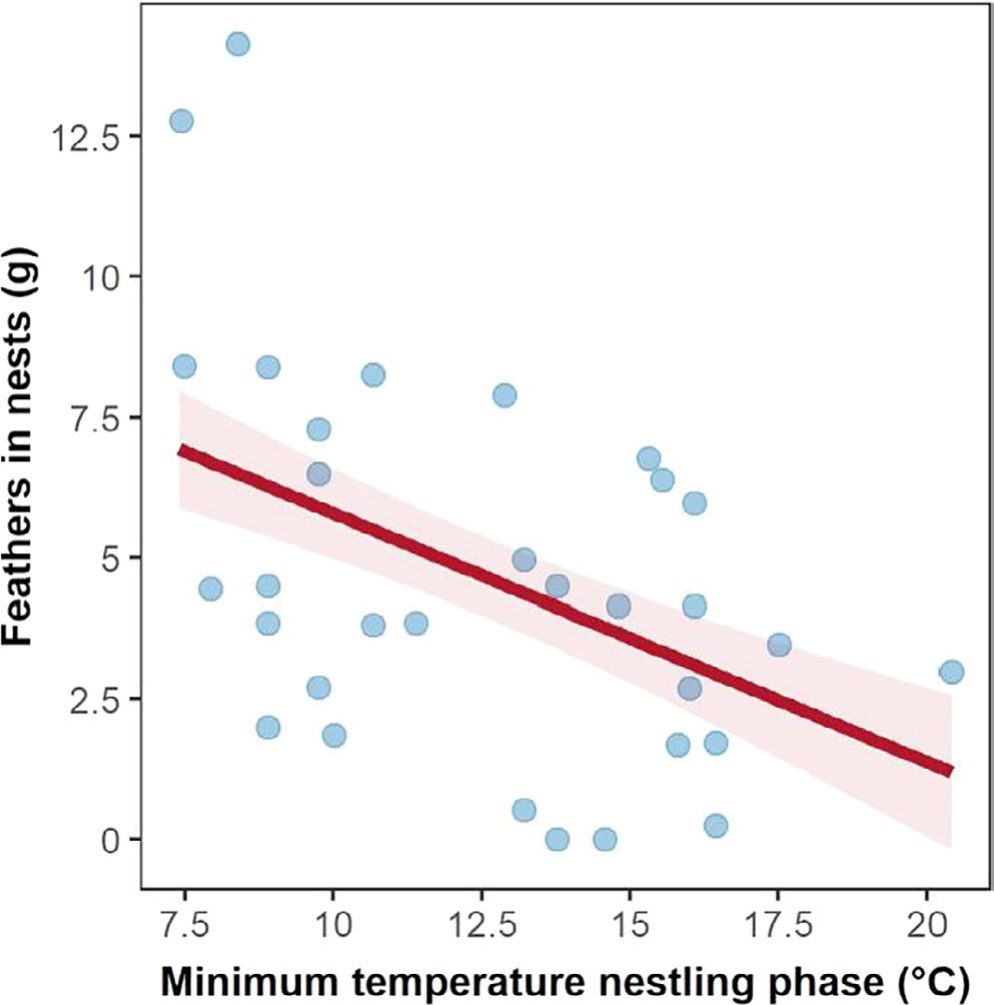
Nests contained more feathers when minimum temperatures during the first ten days of life of tree sparrow nestlings were low. Mean ± *SE* (standard error) model predictions are illustrated in red (based on top model in Table 2), with raw data point represented by transparent blue dots

Overall, nests contained 5.71 g (*SD* = 3.56 g) of feathers (i.e., 8% of dry nest weight), with similar amounts across the two years of study (Table S1). Nests contained more feathers when minimum temperatures during nestling development were low (Table 2; Figure 3). Despite a moderate correlation between minimum temperatures during nestling development and clutch completion dates, the effect of minimum temperature during development received much stronger statistical support than the effect of clutch completion date in predicting the amount of feathers in nests (Table 2).

**TABLE 2:** Nests contained more feathers when minimum temperatures were low

Intercept	Clutch completion date	Minimum temperature (nestling phase)	Rainfall (nestling phase)	Breeding year^a^	*k*	AIC	ΔAIC
0.011		−0.461			5	91.7	0.0
0.042	−0.325				5	95.4	3.6
0.022			0.309		5	95.6	3.9
−0.009				−0.274	5	95.7	4.0
0.029					4	97.1	5.4

Note

The amount of feathers in nests was negatively predicted by minimum temperatures during the nestling rearing phase of reproduction. Models within a ΔAIC value of six retained after applying the nesting rule (Richards, 2008) are shown. Model coefficients are mean‐centered and scaled by one standard deviation. *N* = 32 first broods. *k* = number of model parameters. “Site” and “nest‐box ID” were included as random intercepts. Predictors that did not appear in any model within a ΔAIC value of six after applying the nesting rule are not presented in this table (see methods for a full list of predictors included in these models). Top model explained 44.31% of the total variation in the amount of feathers in nests (i.e., conditional *R* ^2^ ‐ (Nakagawa & Schielzeth, 2013).

^a^ Estimate for the 2012 breeding year.

The amount of feathers in nests did not predict the number of nestlings successfully fledged in our population of tree sparrows (Table S2). Our analysis revealed no effects of nesting feathers on nestling survival, either in isolation or in interaction with environmental conditions (Table S2). Similarly, the number of eggs that survived to hatching was not predicted by the amount of feathers in nests. Clutch size again was the only variable found to strongly determine the number of hatchlings per brood. These results did not significantly change when only first broods were considered (*N* = 32 broods).

## 4 DISCUSSION

Nests are key structures for avian reproduction in altricial birds. As well as providing a physical support for eggs and their incubation, nests can function as a protection against detrimental fluctuations in environmental conditions for developing individuals (Ar & Sidis, 2002; Deeming, 2016). In our urban population of tree sparrows, we found a strong negative effect of rainfall on the number of nestlings that fledged per brood, but this negative effect was not mitigated by the amount of feathers added by breeding adult birds to their nests. We did not find evidence suggesting that the amount of feathers, a material with insulation properties, was associated with improved egg survival to hatching or nestling survival to fledgling. The amount of feathers in nests was negatively associated with the minimum temperatures experienced by nestlings during development; however, variation in minimum temperatures during incubation or nestling development did not impact reproductive performance.

Persistent rainfall during the development of nestlings could have negative effects on nestling survival through several mechanisms. Rainfall could have a direct negative effect on the survival of nestlings if it soaks them, increasing their thermoregulatory demands (Kennedy, 1970; Lustick & Adams, 1977; Webb & King, 1984; Wilson et al., 2004). Although this direct negative effect of rainfall could have contributed toward decreasing the overall survival of nestlings in our population of tree sparrows, its contribution was likely of minor importance given that the study species nested in artificial nest boxes (e.g., Wesolowski et al., 2002). If rainfall had entered nest boxes and directly affected developing nestlings, we may have expected a similar negative effect on egg survival (which we did not find), possibly via indirect negative effects on the incubating individual (Coe et al., 2015). Rainfall can also have a negative impact on nestling survival via indirect effects on parental foraging behavior. For example, the feeding rate to offspring decreased in rainy days in small tree finches (*Camarhynchus parvulus;* Heyer et al., 2020) and great tits (Parus major; Radford et al., 2001). A similar pattern was found in northern wheatears (*Oenanthe oenanthe;* Öberg et al., 2015) and gray catbirds (*Dumetella carolinensis;* Johnson & Best, 1982), with rainfall decreasing nest visitation rates by adults and, in turn, nestlings survival (Öberg et al., 2015). Feeding rates of adults to offspring were not recorded in our study, but they could well represent the main mechanism through which rainfall negatively impacted nestling survival in our urban population of tree sparrows.

By reducing energy allocation of nestlings to thermoregulation during rainfall periods, the amount of feathers in nests could alleviate the negative consequences of rainfall for nestling survival. However, we found no evidence for that prediction as negative rainfall effects on the number of nestlings that survived to fledging did not depend on the amount of feathers in nests. Thermal insulation offered by feathers decreases when feathers are wet (Hilton et al., 2004) and, therefore, it is likely that the benefits of using feathers as nest materials may disappear or be reduced when rainfall occurs. Finding that the amount of feathers in nests was associated with minimum temperatures during nestling development contrasts with the lack of an effect of this nest material on nestlings and egg survival. Associations between the amount of nest cup lining, of which feathers are an important component, and spring temperatures have been found in blue tits (*Cyanistes caeruleus*) and great tits (*Parus major*) (Deeming et al., 2012; Mainwaring et al., 2012). In such observational studies, early breeders, that experienced colder temperatures, built nests with more lining material than late breeders, as observed here for tree sparrows. In an experimental manipulation of long‐tailed tit (*Aegithalos caudatus*) nests, McGowan et al. (2004) showed that the amount of feathers in nests decreased along the breeding season but experimentally adding feathers to nests did not increase the total amount of feathers in nests and, importantly, did not affect the insulation properties of nests (McGowan et al., 2004). That study indicates low natural variation in the insulation properties of nests despite high variation in the amount of feathers present in nests (McGowan et al., 2004). It is possible, then, that by adjusting the amount of feathers in nests according to environmental temperatures, tree sparrows reduced natural variation in the insulation properties of nests (i.e., keeping nest microclimate within a narrow optimal range) and, hence, the lack of a positive effect of feathers in nests on egg and nestling survival reported here (but see Winkler, 1993). An experimental manipulation of the amount of feathers in nests and nest microclimate would help consolidate our findings that nesting feathers do not improve egg or nestling survival in our population of tree sparrows.

The lack of a positive effect of the amount of nesting feathers in the reproductive success of tree sparrows is in line with experimental studies that showed no relationship between the supplementation of feathers to nests and egg or nestling survival (Dawson et al., 2011; Jarvinen et al., 2017; McGowan et al., 2004). However, by creating subtle changes in nest microclimate, feathers in nests could also improve the body condition of nestlings (Dawson et al., 2005; Lombardo et al., 1995). Our data do not allow us to investigate this possibility, but we cannot discard that natural variation in the amount of feathers in tree sparrow nests was associated with morphological or body condition differences among offspring. The absence of an effect of feathers in nests on the reproductive success of tree sparrows in this study could also be explained by at least two other alternative explanations. First, when nest composition is assessed at the end of the breeding season, the signal of an association between nesting feathers and breeding success may have been weakened if adult tree sparrows extract nest materials from preexisting nests of other tree sparrow pairs (as it happens in other species—e.g., Slager et al., 2012). Second, this negative result may be explained by low variation in temperatures due to living in an urban habitat (Arnfield, 2003). In this context, it is conceivable that the potential benefits of bringing feathers to nests are reduced in cities if urban birds are exposed to milder environmental conditions than nonurban bird populations.

In conclusion, this study provides evidence for negative effects of rainfall on reproduction in an urban cavity‐nesting bird population, similarly to those detrimental rainfall effects reported for other species (Heyer et al., 2020; Öberg et al., 2015; Radford et al., 2001; Schöll & Hille, 2020). Additionally, we show that nest composition (i.e., the amount of feathers in nests) did not mitigate these strong negative effects of rainfall on nestling survival; nor did it modulate any effect of temperature on egg or nestling survival. These results suggest that the use of feathers as a nest material has limited consequences for how offspring survive inside nests and cope with short‐term fluctuations in environmental conditions.

## CONFLICT OF INTEREST

The authors declare that there is no conflict of interest.

## AUTHOR CONTRIBUTION

**Pablo Capilla‐Lasheras:** Conceptualization (lead); Data curation (lead); Formal analysis (lead); Funding acquisition (equal); Investigation (lead); Methodology (lead); Project administration (supporting); Visualization (lead); Writing‐original draft (lead); Writing‐review & editing (lead). **Blanca Bondía:** Conceptualization (supporting); Data curation (supporting); Investigation (supporting); Writing‐review & editing (supporting). **Jose Ignacio Aguirre:** Conceptualization (supporting); Funding acquisition (equal); Project administration (lead); Resources (lead); Supervision (lead); Writing‐review & editing (supporting).

## Supporting information

Appendix S1

## Data Availability

The datasets generated and analyzed during the current study are available at the Dryad Digital Repository: https://doi.org/10.5061/dryad.c59zw3r6g
