# Supplementary material for: Environmental conditions but not nest composition affect reproductive success in an urban bird
Source: Ecol Evol. 2021 Mar 12;11(7):3084–92. doi: 10.1002/ece3.7234 (PMC8019055; doi:10.1002/ece3.7234)
Supplement: Supplementary file 1 — Appendix S1 [file ECE3-11-3084-s001.docx]

**APPENDIX:**

**Table S1.** Summary of nest composition for 66 tree sparrow nests collected in the breeding seasons of 2012 and 2013. Nests were dissected and three different nest materials were classified and weighed: feathers, anthropogenic materials and other organic materials (mostly grass). SD = standard deviation; N = sample size.

|  | **Feathers (g)** | | **Anthropogenic materials (g)** | | **Other organic materials (g)** | | | **N** |
| --- | --- | --- | --- | --- | --- | --- | --- | --- |
|  | ***Mean*** | ***SD*** | ***Mean*** | ***SD*** | | ***Mean*** | ***SD*** |  |
| **2012** | 4.87 | 3.19 | 0.74 | 1.12 | | 65.50 | 23.00 | 31 |
| **2013** | 6.46 | 3.74 | 1.41 | 1.77 | | 62.80 | 20.80 | 35 |
| **Both years** | 5.71 | 3.56 | 1.09 | 1.53 | | 64.09 | 21.76 | 66 |

**Table S2**. Effects of environmental conditions and nesting feathers on the number of fledged nestlings. The findings of this analysis are similar to those presented in Table 1, suggesting an important negative effect of the amount of rainfall fallen during the nestling phase (i.e. total rainfall in the first ten days of a nestlings’ lives) on the number of nestlings that fledged. Models within a ΔAIC value of six retained after applying the nesting rule (Richards, 2008) are shown. Model coefficients are mean centered and scaled by one standard deviation. N = sample size; k = number of model parameters. ‘Site’ and ‘nest-box ID’ were included as random intercepts. Predictors that did not appear in any model within a ΔAIC value of six after applying the nesting rule are not presented in this table (see methods for a full list of predictors included in these models; e.g. amount of feathers in nests and its interactions with environmental conditions).

| **Number of fledged nestlings (N = 66 broods)** | | | | | | | | | | | | |
| --- | --- | --- | --- | --- | --- | --- | --- | --- | --- | --- | --- | --- |
| **Intercept** | **Rainfall (incubation)** | **Rainfall (nestling phase)** | **Minimum temperature (incubation)** | **Minimum temperature (incubation)^2^** | **Minimum temperature (nestling phase)** | **Minimum temperature (nestling phase)^2^** | **Clutch size** | **Clutch completion date** | **Clutch completion date^2^** | **k** | **AIC** | **ΔAIC** |
| 1.077 |  | -0.201 |  |  |  |  | 0.124 |  |  | 3 | 254.3 | 0.0 |
| 1.079 |  | -0.338 |  |  | -0.171 |  |  |  |  | 3 | 255.3 | 1.1 |
| 1.085 |  | -0.205 |  |  |  |  |  |  |  | 2 | 255.6 | 1.3 |
| 1.072 | 0.303 |  |  |  |  |  | 0.118 | 0.279 | -0.303 | 5 | 256.4 | 2.1 |
| 1.078 |  |  |  |  | 0.096 | -0.166 | 0.119 |  |  | 4 | 256.6 | 2.3 |
| 1.079 | 0.288 |  |  |  |  |  |  | 0.256 | -0.318 | 4 | 257.0 | 2.7 |
| 1.085 |  |  |  |  | 0.091 | -0.180 |  |  |  | 3 | 257.4 | 3.1 |
| 1.081 |  |  |  |  |  |  | 0.112 | 0.045 | -0.165 | 4 | 257.6 | 3.3 |
| 1.087 |  |  |  |  |  |  |  | 0.034 | -0.184 | 3 | 258.0 | 3.7 |
| 1.095 |  |  |  |  |  |  | 0.135 |  |  | 2 | 258.8 | 4.5 |
| 1.085 | 0.394 |  | 0.409 | -0.276 |  |  |  |  |  | 4 | 259.3 | 5.0 |


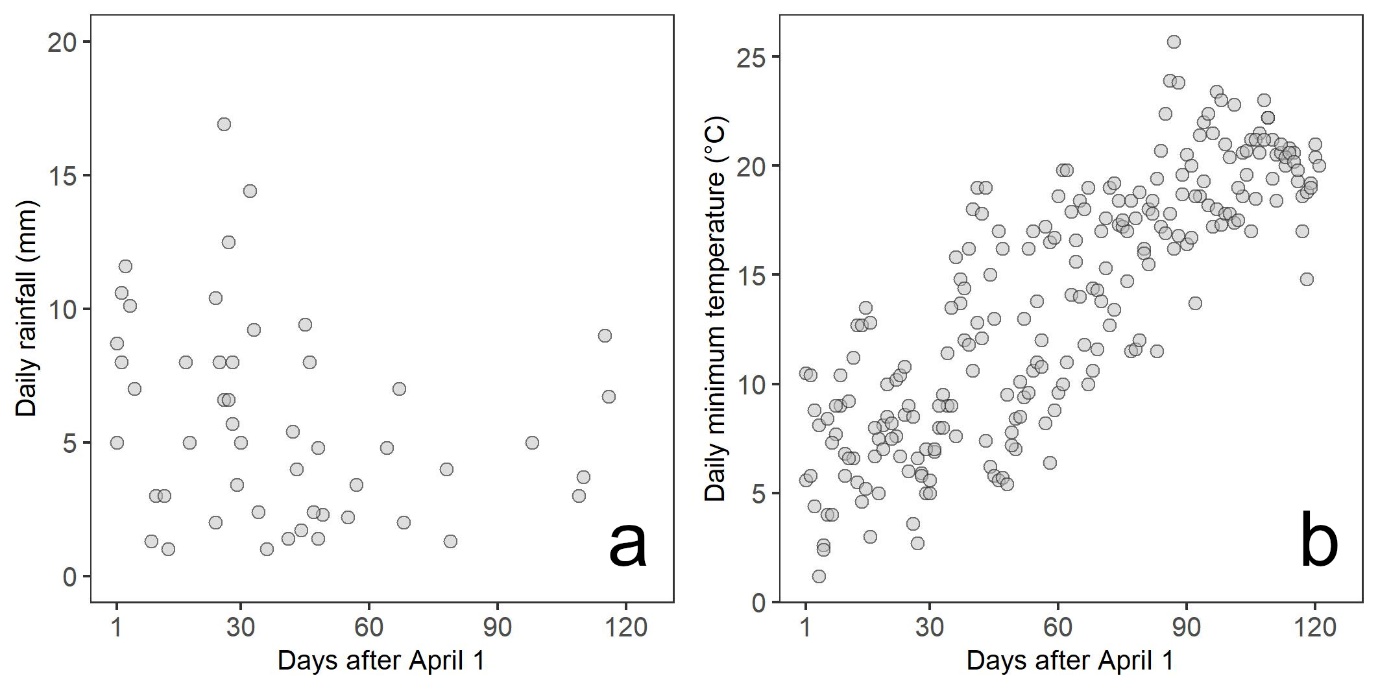


**Figure S1**. Weather conditions throughout the breeding season (two breeding seasons of study combined): (**a**) daily total rainfall and (**b**) daily minimum temperature. Data were extracted from the European Climate Assessment & Data set (http://eca.knmi.nl/) for Retiro, Madrid weather station (40º 24' 55'' N, 3º 41º 03'' W).
